# Pericardial Window Formation Complicated by Intrapericardial Diaphragmatic Hernia

**DOI:** 10.1155/2014/132170

**Published:** 2014-03-03

**Authors:** Jeremy Docekal, Thomas Fabian

**Affiliations:** ^1^Department of Internal Medicine, Tripler Army Medical Center, 1 Jarrett White Road, Honolulu, HI 96859, USA; ^2^Thoracic Surgery Section, Albany Medical Center, 47 New Scotland Avenue, Albany, NY 12208, USA

## Abstract

In rare circumstances, a diaphragmatic defect may allow for herniation of intra-abdominal contents into the pericardial space. These occurrences are exceedingly rare and may be due to trauma or congenital defects of the septum transversum or as the result of surgical procedures. We describe a 73-year-old female who presented with cardiac and abdominal symptoms one month after undergoing a subxiphoid pericardioperitoneal window for treatment and evaluation of a symptomatic pericardial effusion.

## 1. Introduction

We report a case of intrapericardial herniation occurring one month after pericardioperitoneal window formation. We further discuss the controversies surrounding the choice of therapy in the treatment of pericardial tamponade.

## 2. Case Report

The first reported case of intrapericardial diaphragmatic hernia was published in 1903, which was congenital in origin [1]. The vast majority of these cases are due to blunt trauma of the chest or abdomen [2]. In comparison to trauma, iatrogenic causes of intrapericardial herniation are exceedingly rare [2, 3]. Surgical procedures which have been complicated by the herniation of intra-abdominal contents into the pericardial cavity include coronary artery bypass grafting, subxiphoid epicardial pacemaker insertion, and after-creation of a pericardial window [3]; see Figure 2.

A 73-year-old female, with a history of renal transplant occurring in 2008, developed shortness of breath and fatigue while on immunosuppressive agents. The patient was subsequently found to have a moderate sized pericardial effusion with tamponade physiology by echocardiography and large bilateral pleural effusions. The woman underwent subxiphoid pericardioperitoneal window formation with the subsequent extraction of approximately 400 mL of free flowing serous fluid. In addition, the patient underwent bilateral pleural drainage via chest tubes placed during the same procedure. The patient tolerated the procedure well with symptomatic relief and was discharged home without complication.

One month after pericardial window formation, the patient presented to the emergency room with acute epigastric abdominal pain. The patient also described a nonproductive cough, mild shortness of breath, obstipation, and vomiting.

At the time of admission, the patient's vital sounds were notable for decreased pulse pressure. A systolic ejection murmur was auscultated on cardiac exam. Pulmonary examination demonstrated adventitious inspiratory breath sounds, dullness to percussion, and egophony at the right lung base. Additionally, the patient's abdomen was distended, with mild guarding and absent bowel sounds.

**Figure 1:**
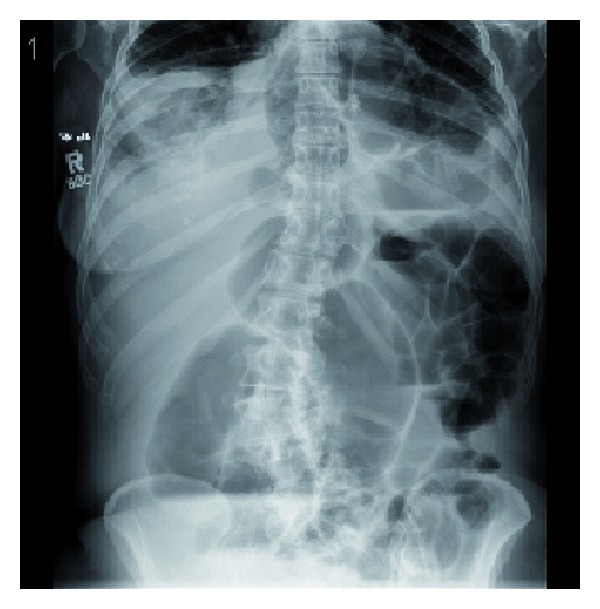
Abdominal radiograph reveals air distended loops of descending and transverse colon. Additionally, a loop of bowel may be seen extending into the pericardial space.

**Figure 2:**
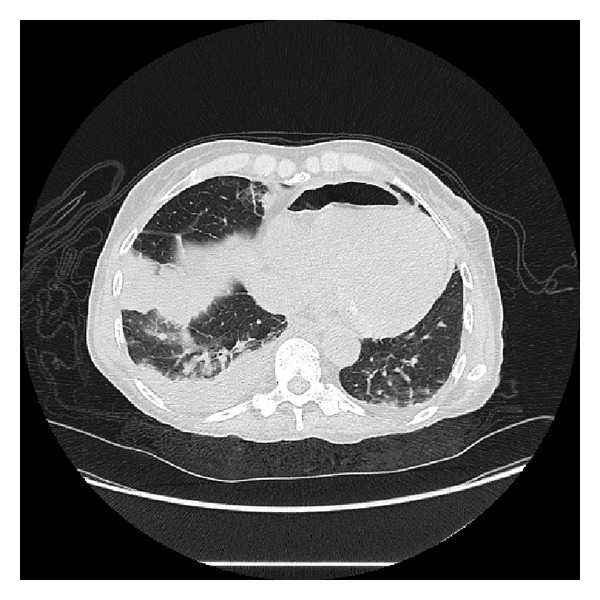
Thoracic CT reveals a loop of bowel incarcerated into the pericardial cavity. This image can be easily mistaken for pneumopericardium secondary to postoperative changes. Right lower lobe consolidation may be appreciated as well.

Admission laboratory studies demonstrated leukocytosis of 25.1 (84% neutrophils), sodium of 130, and elevated BUN/creatinine ratio. Urine osmolarity was elevated at 592, and fractional excretion of sodium was calculated to be less than 1%. Arterial blood gas analyses, performed one day after admission, showed a PH of 7.18, PaO2 of 85 mmHg, and a bicarbonate level of 18. A CT scan of the abdomen demonstrated an intrapericardial hernia which included a portion of the transverse colon; see Figures 1 and 2.

An abdominal laparotomy was performed under general anesthesia for evacuation of the herniated bowel. Upon entry into the abdominal cavity, the first thing that was noted was a dilated colon. The colon was traced up to the site of the pericardial window and the central tendon of the diaphragm and was subsequently reduced out of the pericardial sac. Fortunately, there was no evidence of compromise to the colon that had been incarcerated within the pericardial sac. The remainder of the colon was inspected and found dilated but otherwise unremarkable. The colon was decompressed, and the pericardial window was closed with interrupted figure-of-eight sutures.

The remainder of the patient's hospital course was uneventful, and the patient's bowel function made a full recovery. Fortunately, the patient's pericardial effusion has not reaccumulated, to date.

## 3. Discussion

The techniques employed in the treatment of pericardial tamponade include percutaneous drainage or surgical subxiphoid approaches with either pericardiotomy or the formation of a pericardioperitoneal window. Considerable controversy exists as to the preferred technique amongst the available options [4].

Head to head comparison between the subxiphoid and percutaneous techniques suggests that in terms of safety and efficacy the subxiphoid may be the preferred approach. This is evidenced by a study published by Allen et al. which reveals that the subxiphoid pericardiostomy had a complication rate of 1.1% and no operative deaths. In contrast, percutaneous drainage had significantly higher mortality and complication rates (4% (1 of 23) and 17% (4 of 23), resp.) [5]. These results were confirmed by Susini et al. who noted that the subxiphoid approach was performed in their series without major complication and provided the advantage of being a superior diagnostic procedure, as compared with the percutaneous approach [6]. While these results help to highlight the advantage of the subxiphoid approaches over the percutaneous technique, the subxiphoid technique is not without flaw.

In the subxiphoid approach, a pericardial window is formed, and the pericardial fluid is subsequently drained via either an external drainage system or through the formation of a pericardioperitoneal window. When compared to the external drainage system, the pericardioperitoneal window is associated with decreased instances of postoperative infection and rates of recurrence [4, 7].

Given these benefits, a pericardioperitoneal window was created in this case. This choice is what ultimately led to the development of this rare complication of pericardial hernia. The reason why this patient would develop this complication as opposed to others is not clear. The possibilities include creation of too large window, a relatively small left hepatic lobe which typical would separate the abdomen contents from the window, and ascites. It is the surgeon's opinion that the window was larger than most and this occurred when directing placement of the bilateral chest tubes into the pleural spaces.

In contrast to the subxiphoid and percutaneous methods, which have numerous comparative studies, there are very few studies comparing the pericardioperitoneal window technique with either the subxiphoid and percutaneous methods. However, the published literature supports the pericardioperitoneal technique as a safe and effective treatment of pericardial effusion.

This case report illustrates that pericardial hernias can occur following creation of pericardioperitoneal communication and clinicians must be aware of this complication to allow appropriate intervention.
